# Assessment of SARS-CoV-2 Mu Variant Emergence and Spread in Colombia

**DOI:** 10.1001/jamanetworkopen.2022.4754

**Published:** 2022-03-30

**Authors:** Juan Hernandez-Ortiz, Andres Cardona, Karl Ciuoderis, Francisco Averhoff, Maria-Angelica Maya, Gavin Cloherty, Jorge E. Osorio

**Affiliations:** 1 Laboratorio Genómico One Health, Colombia-Wisconsin One Health Consortium, Universidad Nacional de Colombia, Medellín, Antioquia; 2 Infectious Disease Research, Abbott, Abbott Park, Illinois; 3 Secretaria Seccional de Salud de Antioquia, Hospital San Vicente Fundación, Medellín, Colombia; 4 Department of Pathobiological Sciences, University of Wisconsin, Madison

## Abstract

This cross-sectional study assesses the emergence and spread of the SARS-CoV-2 Mu variant compared with other variants in Antioquia State, Columbia, from July 2020 to August 2021.

## Introduction

Since it emerged in Colombia in January 2021, the SARS-CoV-2 Mu variant has spread to 34 countries. The epidemiology of this variant has not yet been fully described. Here we report on the emergence and spread of the Mu variant in Antioquia State, Colombia.

## Methods

This cross-sectional study was approved by the Corporación para Investigaciones Biológicas Institutional Review Board. Participants provided written informed consent. The study followed the Strengthening the Reporting of Observational Studies in Epidemiology (STROBE) reporting guideline.

From July 15, 2020, to August 31, 2021, nasopharyngeal swabs or bronchoalveolar wash samples were obtained from patients with suspected SARS-CoV-2 infection who presented to health institutions in Antioquia State, Colombia. Deidentified demographic and clinical data were collected via self-report, including patient age, sex, clinical outcomes, and risk factors.

SARS-CoV-2 infection was confirmed with reverse transcription polymerase chain reaction, and the whole genome was sequenced for patient samples with a cycle threshold value of 27 or less.^1^ Phylogenetic analysis was conducted using Pangolin software.^2^ Genome sequences obtained in this study were deposited in the GISAID (Global Initiative on Sharing Avian Influenza Data) database.^3^ Characteristics of persons with Mu infection were compared with those of individuals with other circulating SARS-CoV-2 variants. We used a nonparametric χ^2^ test to compare differences between groups; *P* < .05 was considered significant. Statistical analysis was performed with R software, version 4.1.2 (R Foundation for Statistical Computing).

## Results

From July 2020 through August 2021, 1032 viral samples were obtained for whole-genome sequencing. Demographic and risk factor data were available for 863 participants (83.4%). Of these individuals, 432 (50.1%) were female and the mean (SD) age was 42.4 (20.9) years (Table); 97 (11.2%) reported being vaccinated against COVID-19.

**Table.** Characteristics of Participants With the SARS-CoV-2 Mu Variant and Other Variants in Colombia, From July 2020 to August 2021

Characteristic	Total, No. (%)	Mu variant, No. (%)	All other variants^a^		VOCs^b^		Non-VOCs^c^	
			No. (%)	*P* value^d^	No. (%)	*P* value	No. (%)	*P* value
No. of patients (%)	863 (100)	283 (32.8)	580 (67.2)	NA	341 (39.5)	NA	239 (27.7)	NA
Age, y^e^								
≤17	79 (9.2)	20 (7.1)	59 (10.2)	.25	39 (11.4)	.07	20 (8.4)	.58
18-29	176 (20.4)	62 (21.9)	114 (19.7)		69 (20.2)		45 (18.8)	
30-39	188 (21.8)	55 (19.4)	133 (22.9)		81 (23.8)		52 (21.8)	
40-49	118 (13.7)	45 (15.9)	73 (12.6)		46 (13.5)		27 (11.3)	
50-59	113 (13.1)	33 (11.7)	80 (13.8)		49 (14.4)		31 (13.0)	
≥60	189 (21.9)	68 (24.0)	121 (20.9)		57 (16.7)		64 (26.8)	
Sex								
Male	431 (49.9)	143 (16.6)	288 (33.3)	.86	165 (48.4)	.65	123 (51.5)	.90
Female	432 (50.1)	140 (16.2)	292 (33.9)		176 (51.6)		116 (48.5)	
Clinical outcome								
Asymptomatic or mild case	636 (73.7)	213 (75.3)	423 (72.9)	.55	269 (78.9)	.53	154 (64.4)	.01
Hospitalized or ICU stay	129 (14.9)	37 (13.1)	92 (15.9)		40 (11.7)		52 (21.8)	
Death	98 (11.4)	33 (11.7)	65 (11.2)		32 (9.4)		33 (13.8)	
Comorbidities^f^								
None	588 (68.1)	201(71.0)	387 (66.7)	.41	236 (69.2)	.87	151 (63.2)	.13
≥1	209 (24.2)	61 (21.6)	148 (25.5)		79 (23.2)		69 (28.9)	
Other	66 (7.6)	21 (7.4)	45(7.6)		26 (7.6)		19 (7.9)	

Abbreviations: ICU, intensive care unit; NA not applicable; VOC, variant of concern.

^a^ Includes Alpha, Delta, Gamma, Lambda, B.1.625, and other lineages.

^b^ Includes Alpha, Delta, and Gamma.

^c^ Includes Lambda, B.1.625, and other lineages.

^d^ Differences among the Mu variant, VOCs, and non-VOCs were tested with the χ^2^ test; *P* < .05 was considered significant.

^e^ The median age was 39 years (range, 0-99 years); the mean (SD) age was 42.4 (20.9) years.

^f^ Comorbidities include risk factors such as diabetes, coronary artery disease, chronic obstructive pulmonary disease, HIV, obesity, chronic kidney failure, hypertension, asthma, cancer, cerebrovascular disease, pregnancy, and smoker status. “Other” indicates conditions not listed here.

The Mu variant was first detected in Antioquia in March 2021. By July 2021, this variant accounted for 52 SARS-CoV-2 genome sequences (85.2%) (Figure, A), coincident with the largest wave of COVID-19 cases reported in Antioquia since the start of the pandemic (Figure, B). The prevalence of the Mu variant decreased to 34 sequences (55.7%) in August 2021, coincident with the introduction of the Delta variant. Except for clinical outcomes, there were no differences in age, sex, or risk factors among persons with Mu infection compared with the other variants circulating at the time (Table).

**Figure.**
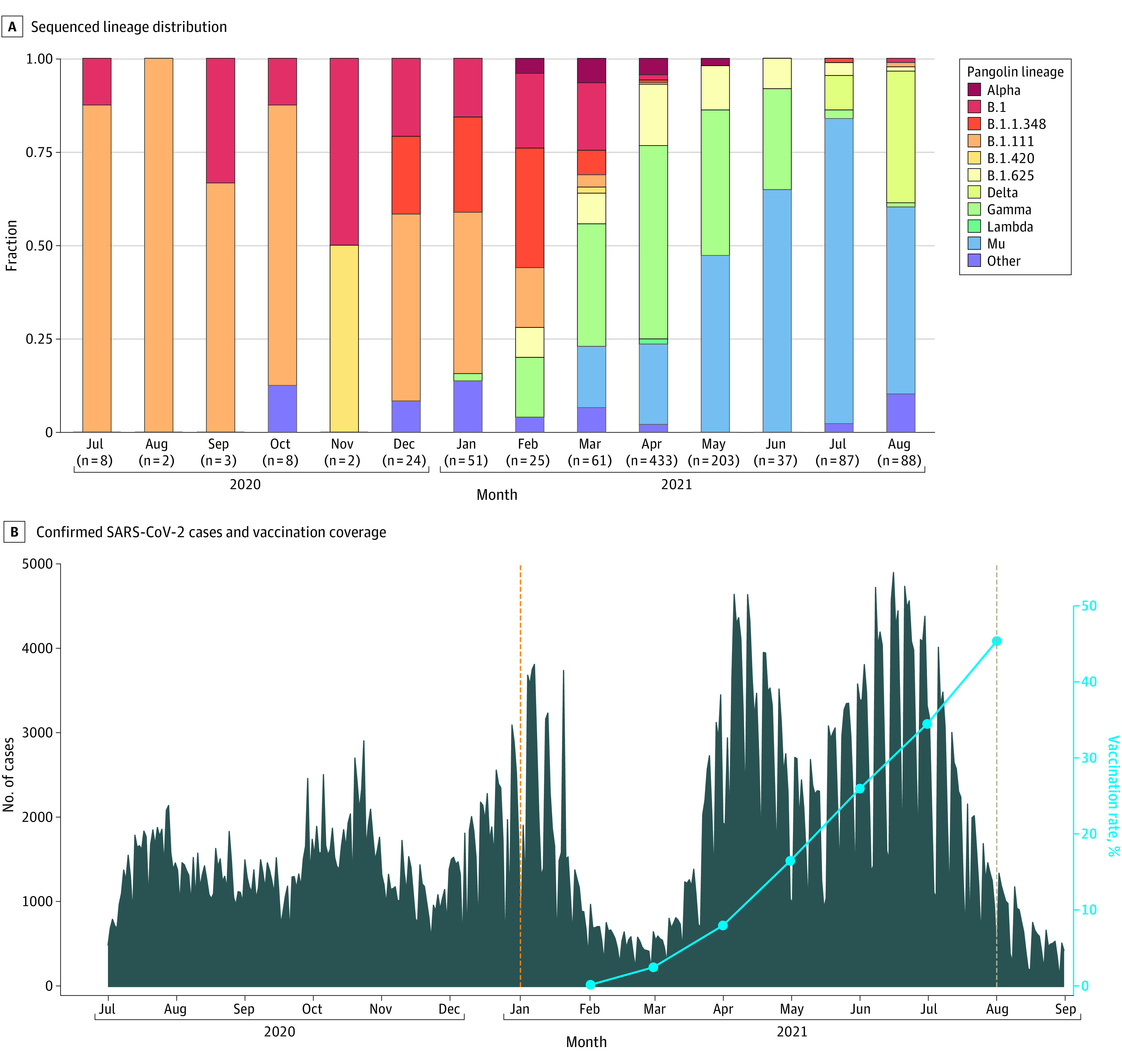
SARS-CoV-2 Sequenced Lineage Distribution, Laboratory-Confirmed Cases, and Vaccination Coverage in Antioquia State, Colombia, From July 2020 to August 2021 A, All 1032 Global Initiative on Sharing Avian Influenza Data reported sequences for Antioquia State, including the 863 sequences obtained in this work. The number of sequences recorded for each month are given in parentheses. B, During March and April 2021, hospital-based monitoring was expanded to include additional ambulatory sites in the state. Dashed lines indicate the first detection dates of the Mu (orange) and Delta (light brown) variants of SARS-CoV-2.

## Discussion

To our knowledge, this is the first study to describe the molecular epidemiology of the Mu variant after it emerged in Colombia in 2021. The large number of cases during the study period is coincident with the introduction of the Mu variant; coupled with low vaccination coverage (45.5% by August, and high levels of SARS-CoV-2 antibody in the population^4^), this observation suggests that reinfection is likely common with the Mu variant. Previous studies have suggested that the Mu variant is resistant to convalescent and vaccine sera.^5,6^ The ability of the Mu variant to evade the immune response in vaccinated and previously infected individuals requires further study.

Our study has some limitations. Because of the insufficient number of vaccinated persons in our study, we were unable to determine whether the Mu variant is more successful at evading resistance from vaccination compared with other variants. The observational nature of this study limits the generalizability of our findings, and the demographic, risk factor, and vaccination data were self-reported. Few persons were vaccinated at the time, and there were several vaccines in use in Colombia; thus, we were not able to assess the effectiveness of vaccination on the Mu variant.

Our data reflect the variant circulation in other populated regions of Colombia.^6^ With the introduction of the Delta and Omicron variants into the country, additional studies are needed to better understand the effect of the Mu variant.
